# The formation of preschooler’s creative personality: the promotion mechanism of positive family routines

**DOI:** 10.1186/s40359-026-04506-5

**Published:** 2026-04-09

**Authors:** Shenghong Dong, Xinyan Li, Yuzhi Yan

**Affiliations:** 1 School of Psychology, Jiangxi Normal University, Nanchang, Jiangxi China; 2 Jiangxi Provincial Philosophy and Social Sciences Laboratory for Data Science and Intelligent Psychological Assessment and Services, Nanchang, Jiangxi China; 3 School of Education, Jiangxi Normal University, Nanchang, Jiangxi China; 4 Jiujiang Vocational University, Jiujiang, Jiangxi, China

**Keywords:** Creative personality, Positive family routines, Autonomy

## Abstract

**Background:**

Preschool is a sensitive period for the formation of creative personality, yet little is known about how everyday family routines contribute to preschoolers’ creative personality or through which psychological mechanisms these effects occur.

**Methods:**

Using classroom‑cluster sampling, 664 preschool children aged 3–6 years and their parents from kindergartens in Jiangxi Province participated. Parents completed three validated questionnaires: the Chinese Family Routines Scale, the Autonomy of Children Scale, and the Creative Personality subscale of the Creativity Assessment Packet. Descriptive statistics and Pearson correlations were computed, followed by structural equation modeling and bias‑corrected bootstrap mediation analyses.

**Results:**

Positive family routines were significantly and positively correlated with autonomy (*r* = .335, *p* < .001) and creative personality (*r* = .339, *p* < .001), and autonomy was positively correlated with creative personality (*r* = .347, *p* < .001). Positive family routines significantly predicted creative personality (γ = 0.412, *p* < .001) and autonomy (γ = 0.505, *p* < .001), and autonomy significantly predicted creative personality (γ = 0.433, *p* < .001). Autonomy partially mediated the association between positive family routines and creative personality, accounting for 53.16% of the total effect.

**Conclusions:**

Positive, structured, and emotionally supportive family routines are positively associated with preschoolers’ creative personality, both directly and indirectly through higher autonomy, highlighting autonomy‑supportive family environments as a key target for early interventions to foster creative development.

**Supplementary Information:**

The online version contains supplementary material available at 10.1186/s40359-026-04506-5.

## Introduction

### Creativity and creative personality in early childhood

Creativity is commonly defined as the capacity to produce ideas or products that are both novel and appropriate or useful within a given context [1, 2]. Recent work has emphasized that creativity involves not only divergent thinking and problem solving, but also the flexible adaptation of ideas to task constraints and social expectations, as well as the evaluative processes that determine which ideas are worth pursuing [1–4]. At a more integrative level, classic frameworks such as the “4Ps” model (person, process, product, and press) [5] and the investment theory of creativity [6] highlight that creativity arises from the confluence of individual characteristics, cognitive processes, products, and environmental press. In line with these perspectives, the present study focuses primarily on the “person” and “press” dimensions, examining how children’s creative personality (person) is shaped within the family environment (press) [6, 7].

Creative personality was first introduced by Guilford [8] as the set of personality characteristics that promote and sustain creative development and the completion of creative tasks [9]. In contemporary work, creative personality is typically defined as a constellation of relatively stable dispositional characteristics—such as curiosity, openness to experience, risk‑taking, and perseverance—that support the generation and implementation of creative ideas across time and situations [8–10]. Following Lin’s framework [9], creative talent can be conceptualized as the joint functioning of creative thinking (primarily cognitive processes) and creative personality (primarily non‑cognitive dispositions). Within this framework, creative personality is viewed as a core, relatively stable component that channels creative potential into actual creative achievement over time [10].

A substantial body of research further suggests that creativity and creative personality are closely tied to positive affect, self‑realization, and psychological well‑being [11–14]. From a sociocultural perspective, however, creativity is not universal in its conception or expression. Cross‑cultural research shows that Western traditions tend to emphasize novelty, autonomy, and the breaking of conventions, whereas Eastern traditions often place greater weight on social harmony, usefulness, and contributions to collective goals when evaluating what counts as creative [15, 16]. Because the present study is conducted in a Chinese context while drawing heavily on theoretical frameworks developed in Western psychology, it is important to situate our work within this cross‑cultural literature and to examine how family “press” may foster creative personality under Eastern sociocultural values [15–18].

### Developmental significance of the preschool period

Compared with school‑age children, adolescents, and adults, preschoolers have received relatively less attention in empirical research on creative personality [19, 20]. However, developmental psychology and educational practice increasingly recognize the preschool years as a sensitive period for the emergence and shaping of creative personality. Studies have shown that ages 2–5 are particularly important for the formation of basic personality structures, and that personality tendencies formed during this period can have long‑lasting effects on children’s later development [8, 21]. During these early years, children’s curiosity, imagination, initiative, and willingness to try new things flourish, and these behavioral tendencies mirror key facets of creative personality [22, 23].

If the cultivation of creative personality is neglected during the preschool years, the foundational dispositions that support creativity may not be fully established, which can in turn constrain creative development across the life span [21, 22, 24]. In contrast, when children are provided with supportive environments that encourage exploration, questioning, and imaginative play, creative personality traits are more likely to be consolidated and strengthened [9, 19, 25]. Given that preschoolers spend most of their time in family and preschool settings, it is crucial to identify the contextual factors in these environments that contribute to the development of creative personality and to clarify the mechanisms through which these factors operate [7, 25]. In addition, examining these processes in non‑Western contexts can help determine whether early environmental supports for creative personality, documented mainly in Western samples, generalize across different sociocultural settings [15, 16].

### Family routines and preschoolers’ creative personality

From a developmental–ecological perspective, creativity arises from dynamic interactions among individual characteristics and environmental contexts [7, 25]. Bronfenbrenner’s ecological systems theory [7] highlights the family as a central microsystem in early childhood, exerting a pervasive influence on children’s cognition, emotion, and personality development [26]. In the preschool years, the family is not only the primary source of basic care and emotional security but also the main arena in which children’s curiosity, exploration, and self‑expression are either supported or constrained [26, 27]. Complementing this ecological view, Bandura’s theory of triadic reciprocal determinism [28] emphasizes that behavior, personal factors (e.g., temperament, cognitive traits, personality), and environmental conditions mutually influence one another. From this perspective, preschoolers’ creative personality can be seen as one aspect of their internal resources that both shapes and is shaped by everyday family routines [17, 28].

Family routines refer to repeated, predictable patterns of family activities and interactions that occur in daily life [26, 29]. However, the term “positive family routines” requires conceptual clarification. Traditional views and some empirical work on rigid rule enforcement have sometimes treated routines and creativity as almost incompatible, arguing that strict rules and highly structured environments may suppress children’s spontaneous exploration and divergent thinking [30–34]. In such work, family routines are often equated with constraining routines, characterized by unilateral parental authority, rigid obedience to expectations, and limited opportunities for child initiative. Under these conditions, children may have fewer chances to express their ideas, make choices, or experiment with alternative solutions, which in turn may hinder the development of creative personality.

More recent perspectives, however, distinguish between constraining routines and positive, autonomy‑supportive routines [26, 35, 36]. Families do not simply impose routines on children; rather, they can co‑construct stable yet flexible patterns of daily activities that combine predictability with emotional warmth and meaningful participation. Drawing on Zhang [37] and Spagnola and Fiese [26] we conceptualize positive family routines as predictable, stable, and jointly endorsed patterns of family activities that provide structure and emotional security while also respecting children’s needs, voices, and age‑appropriate agency [26, 35, 37]. In the present study, positive family routines are operationalized through four dimensions of the Chinese Family Routines Scale—child education routines, leisure routines, celebratory routines, and interactions with extended family—which together index frequent, emotionally warm, and organized family practices in everyday life [37]. These routines can offer rich opportunities for conversation, joint problem solving, and shared decision making, all of which may foster the curiosity, openness, and persistence characteristic of creative personality [9, 19, 25].

Distinguishing positive family routines from constraining routines is essential for clarifying how the family environment relates to preschoolers’ creative personality [26, 35, 37]. Whereas constraining routines are marked by unilateral control, rigid rule enforcement, and limited child agency, positive family routines are characterized by shared expectations, emotional closeness, and opportunities for children to participate in daily decision making [26, 35, 36]. Building on this distinction, we hypothesize that higher levels of positive family routines—rather than routines per se—will be associated with higher levels of preschoolers’ creative personality.

### Autonomy as a mediator between family routines and creative personality

Autonomy in early childhood refers to children’s capacity to act independently, regulate their behavior and emotions, and make age‑appropriate choices in daily life [26, 38]. Guilford [8] already highlighted autonomy as a core facet of creative personality, and subsequent work has linked autonomous functioning to flexible, self‑initiated problem solving and creative engagement [39, 40]. From a developmental perspective, the preschool years constitute a critical period for the emergence of autonomy, during which children gradually learn to separate from caregivers, take responsibility for simple tasks, and express their own preferences and opinions [24, 38].

Bronfenbrenner’s ecological systems theory suggests that proximal processes in the family microsystem play a central role in fostering or hindering children’s autonomy [7]. Family routines are an important part of these proximal processes. When routines are predictable, stable, and emotionally supportive, they can provide a secure structure within which children are encouraged to make choices, try new activities, and learn self‑regulation skills [26, 27, 37]. In contrast, rigid and highly controlling routines may restrict children’s opportunities to practice decision making and independent problem solving, thereby impeding the development of autonomy [30, 34].

Self‑Determination Theory (SDT) further provides a motivational framework for understanding why autonomy is important for creativity [41]. According to SDT, autonomy‑supportive environments—where children’s perspectives are acknowledged, they are offered meaningful choices, and external pressures are minimized—facilitate intrinsic motivation, exploratory behavior, and creative engagement [25, 41]. Recent work also suggests that autonomy‑supportive processes can foster compensatory creative development even in less favorable family contexts, underscoring the proximal role of autonomy in linking family environment to creativity [42].

Integrating these perspectives, we propose a mediation mechanism in which positive family routines shape preschoolers’ creative personality indirectly via autonomy. When family routines are structured yet flexible, and when parents invite children’s input in everyday activities (e.g., choosing games, helping with household tasks, or planning family outings), children are more likely to experience themselves as competent, self‑directed agents. This sense of autonomy, in turn, may promote creative personality traits such as curiosity, initiative, persistence in the face of difficulties, and openness to new experiences [17, 40]. Consistent with Bronfenbrenner’s ecological systems theory [7], Bandura’s triadic reciprocal determinism [28], and Self‑Determination Theory [41], autonomy thus provides a theoretically grounded pathway through which positive family routines (press) may influence children’s creative personality (person) over time.

### The present study

Despite growing interest in the role of family context in creativity, empirical work on preschoolers’ creative personality—and on how everyday family routines shape it—remains limited, particularly in non‑Western cultural settings such as China [19, 20, 22]. Most prior studies have focused either on cognitive aspects of creativity (e.g., divergent thinking) or on older age groups, with relatively few investigations integrating family routines, autonomy, and creative personality within a unified developmental framework [3, 4, 36]. Grounded in Bronfenbrenner’s ecological systems theory, Bandura’s triadic reciprocal determinism, and SDT [7, 28, 41], the present study adopts a developmental perspective to examine how positive family routines and autonomy jointly contribute to preschoolers’ creative personality in a Chinese context.

First, we investigate the direct association between positive family routines and preschoolers’ creative personality. Based on prior work distinguishing supportive from constraining routines and emphasizing the developmental salience of early family environments [25, 26, 35, 37], we hypothesize that higher levels of positive family routines will predict higher levels of creative personality (Hypothesis 1).

Second, we test whether children’s autonomy mediates the association between positive family routines and preschoolers’ creative personality. Drawing on ecological, motivational, and sociocultural theories, we propose that positive family routines provide a structured yet autonomy‑supportive context that fosters children’s autonomous functioning, which in turn supports the development of creative personality [17, 28, 40, 41]. Accordingly, we hypothesize that autonomy will partially mediate the positive association between family routines and creative personality (Hypothesis 2).

**Fig. 1:**
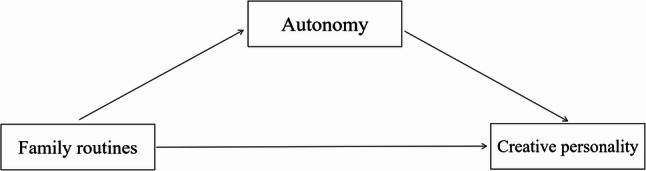
Hypothesized mediation model of autonomy between positive family routines and creative personality

Accordingly, the present study addresses two research questions from a developmental perspective: (1) What is the relationship between positive family routines and preschoolers’ creative personality? (2) Does autonomy play a mediating role in this relationship? By focusing on the preschool years and on everyday family routines, this study aims to enrich developmental and cross‑cultural understandings of how home environments support the emergence of creative personality and to provide a theoretical basis for designing family‑ and school‑based interventions that foster autonomy and creativity in early childhood. The hypothesized mediation model is illustrated in Fig. 1.

## Methods

### Participants and procedure

Data were collected using cluster random sampling at the classroom level, and participants were recruited from public and private kindergartens in Jiangxi Province, China. This study strictly adhered to relevant laws and regulations in China, and all procedures were noninvasive and observational in nature. The research protocol was reviewed and approved by the Institutional Review Board of the School of Psychology at Jiangxi Normal University, in accordance with the Ethical Guidelines for Psychological Research issued by the Chinese Psychological Society (Approval No. IRB‑JXNU‑PSY‑2021015).

Written informed consent was obtained from parents or legal guardians through the participating kindergartens. Prior to data collection, trained research assistants introduced the study aims, procedures, and confidentiality safeguards to head teachers, who then relayed standardized instructions to parents on how to complete the questionnaires. Parents were given five consecutive days to fill out the questionnaires at home and return them in sealed envelopes to the teachers, after which the experimenters collected all materials.

A total of 707 preschool children initially took part in the study. Following conventional data‑screening procedures, cases with substantial missing data or extreme outliers were excluded, yielding 664 valid questionnaires (352 boys, 312 girls; *M*_age_= 4.12 years, *SD*_age_= 0.92). The effective response rate was 93.92%.

### Measures

#### Family routines scale, FRS

Positive family routines were assessed using the Chinese Family Routines Scale (FRS) developed by Zhang [37]. The scale consists of 21 items covering four dimensions: educational routines (e.g., parents’ involvement in children’s learning and daily care), leisure routines (e.g., family arrangements during shared leisure time), celebratory routines (e.g., celebrations of birthdays and family anniversaries), and extended‑family interaction routines (e.g., regular communication with relatives). Parents rated each item on a 5‑point Likert scale (1 = “completely consistent” to 5 = “completely inconsistent”), with lower raw scores indicating more frequent and stable family routines.

For ease of interpretation, all items were reverse‑coded in the present study so that higher scores reflected a higher level of positive family routines (i.e., more frequent, predictable, and emotionally supportive family activities). Previous research has demonstrated good reliability and validity of the FRS in Chinese samples [43]. In the current sample, Cronbach’s α was 0.90.

#### Autonomy of children scale

Children’s autonomy was measured using the Chinese Autonomy of Children Scale developed by Hei [38], which comprises 22 items tapping three dimensions: behavioral autonomy, emotional autonomy, and cognitive autonomy. Parents rated each item on a 5‑point Likert scale (1 = “never” to 5 = “always”), with higher scores indicating a higher level of autonomy in daily life. In the present study, Cronbach’s α was 0.70, indicating acceptable internal consistency for research purposes.

#### Creativity assessment packet, CAP

Preschoolers’ creative personality was assessed using the Creative Personality subscale of the Chinese version of the Creativity Assessment Packet (CAP) adapted by Lin and Wang [44]. The scale includes 50 items measuring four facets of creative personality: risk‑taking, curiosity, imagination, and challenge. Parents rated each item on a 3‑point Likert scale (1 = “completely inconsistent” to 3 = “completely consistent”), with higher scores reflecting a higher level of creative personality. In the current study, Cronbach’s α was 0.86, indicating good internal consistency.

### Data analysis

All statistical analyses were conducted using IBM SPSS Statistics 23.0 and Mplus 7.0. We first computed descriptive statistics (means and standard deviations) and zero‑order Pearson product–moment correlations among the focal variables. Given that all scales were multi‑item Likert‑type measures with at least three response categories and approximately normal distributions, Pearson correlations were used to summarize the linear associations among variables as an initial step.

To test the hypothesized mediation mechanism, we then used structural equation modeling (SEM) with bootstrap estimation. SEM analyses were conducted in Mplus 7.0 at the observed‑variable level to estimate the direct and indirect paths from positive family routines and autonomy to creative personality and to evaluate overall model fit using standard indices (χ²/df, RMSEA, CFI, TLI, SRMR). Bias‑corrected bootstrap confidence intervals for the indirect effect of positive family routines on creative personality via autonomy, as well as the proportion of the total effect accounted for by this pathway, were obtained directly from the SEM model in Mplus 7.0, based on 5,000 bootstrap resamples.

## Results

### Common method deviation test

Common method bias may exist because all of the data were generated by parent‑report questionnaires, which may decrease the validity of the results. Generally, there are two ways of controlling common method bias, procedural remedies and statistical remedies. Procedural remedies refer to control measures incorporated into the process of a study’s design and measurement by researchers. In this study, we strictly followed principles of confidentiality and voluntariness, and asked parents to truthfully fill out each item in the questionnaire according to their child. The questionnaire was filled in anonymously, and the directions of all questionnaires were standardized and presented in a uniform format, which helped partially control common method bias.

In addition, statistical remedies involve a statistical test that is applied after data collection. A confirmatory factor analysis (CFA) was used to test for common method bias in this study, and the one factor model fitting indexes were χ^2^/df = 3.295, RMSEA = 0.059, CFI = 0.395, TLI = 0.382, and SRMR = 0.078, which indicate poor model fit and thus suggest that there was no serious common method bias in the variables in this study.

### Descriptive statistics

By using SPSS version 23.0, the mean value, standard deviation, and Pearson product- moment correlation coefficients of each variable were computed. As shown in Table 1, the mean value and standard deviation of each variable were within the acceptable range. Positive family routines were significantly positively correlated with autonomy and creative personality (*r* = .335, *p* < .001; *r* = .339, *p* < .001), indicating that higher levels of positive family routines, the higher the level of both preschoolers’ autonomy and creative personality; and autonomy was significantly correlated with preschoolers’ creative personality (*r* = .347, *p* < .001), indicating that the better preschoolers’ autonomous development, the higher their creative personality level. These bivariate correlations provide preliminary support for the hypothesized associations among the variables and justify subsequent mediation analyses using SEM with bootstrap estimation in Mplus 7.0.

**Table 1:** Means, standard deviations, and Pearson correlations among study variables

Variables	1	2	3
1. Family Routines	-		
2. Autonomy	0.335^***^	-	
3. Creative Personality	0.339^***^	0.347^***^	-
M	4.050	3.450	2.127
SD	0.521	0.382	0.222

*N* = 664; Pearson correlations are reported

^***^p < .001

### Structural equation model analyses

**Fig. 2:**
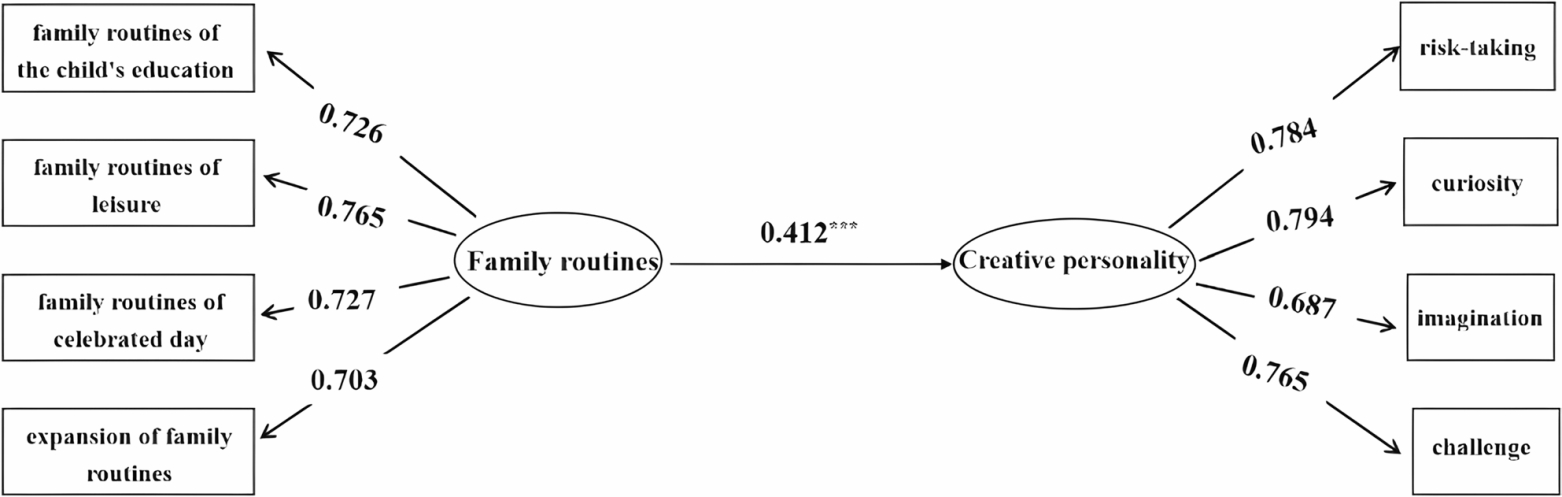
Model 1, the relationship between family routines and creative personality.

Based on the analytic strategy described above, structural equation models were estimated in Mplus 7.0, and indirect effects were evaluated using bootstrap procedures within the SEM framework. First, the main effect was tested, with positive family routines as the independent variable and creative personality as the dependent variable, to construct Model 1 (as shown in Fig. 2). Model 1 showed good fit to the data (*χ*^*2*^*/df* = 1.585, RMSEA = 0.030, CFI = 0.994, TLI = 0.992, SRMR = 0.016). The results indicated that positive family routines significantly predicted creative personality (*γ* = 0.412, *t* = 9.918, *p* < .001), supporting H1. In this model, the standardized total effect of positive family routines on creative personality was 0.412 (SE = 0.040, 95% CI [0.298, 0.509]), which reflects a moderate effect size in practical terms.

**Fig. 3:**
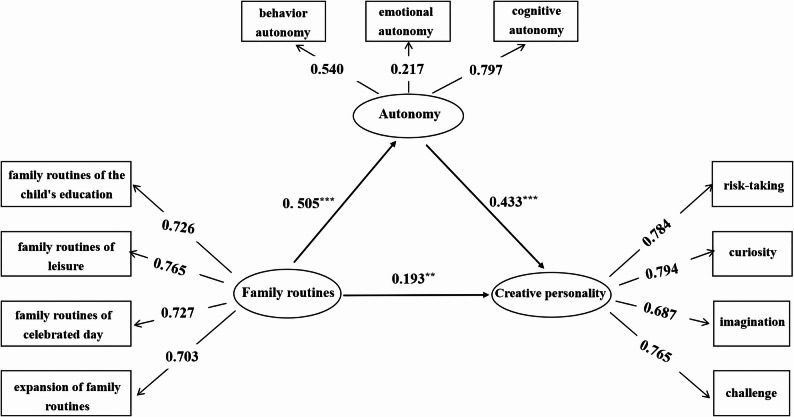
Model 2: Autonomy as a mediator between positive family routines and creative personality

Second, Model 2 was specified by adding autonomy as a mediator between positive family routines and creative personality (as shown in Fig. 3). Model 2 also demonstrated good fit (χ2/df = 1.984, RMSEA= 0.039, CFI= 0.983, TLI= 0.977, SRMR= 0.024). Positive family routines significantly predicted autonomy (γ = 0.505, t = 10.538, p< .001), and autonomy significantly predicted creative personality (γ = 0.433, t = 7.782, p< .001)

Using bootstrapping with 5,000 resamples in Mplus 7.0, we tested the mediating effect of autonomy. The results showed that the indirect effect of positive family routines on creative personality via autonomy was 0.219 (SE = 0.036), with a 95% bias‑corrected confidence interval [0.141, 0.333], which did not include zero, indicating a significant mediation effect. The indirect pathway accounted for 53.16% of the total effect of positive family routines on creative personality, thereby supporting H2. A detailed decomposition of the total, direct, and indirect effects is presented in Table 2.

**Table 2:** Decomposition of the total, direct, and indirect effects of positive family routines on creative personality (bootstrap analysis)

Effect type	Effect	SE	95% CI LL	95% CI UL	Proportion of total effect
Total effect (FR→CP)	0.412	0.040	0.298	0.509	
Direct effect (FR→CP)	0.193	0.056	0.044	0.337	46.84%
Indirect effect effect (FR→AUT→CP)	0.219	0.036	0.141	0.333	53.16%

*FR* positive family routines, *AUT* autonomy, *CP* creative personality

All effects are standardized estimates. Bootstrap estimates are based on 5,000 resamples with bias‑corrected 95% confidence intervals. Proportion of total effect refers to the ratio of each effect to the total effect of FR on CP

## Discussion

### Family routines and creative personality

Consistent with a developmental perspective that views the preschool years as a sensitive period for creative development [22, 23], our structural equation modeling results showed that positive family routines significantly and positively predicted preschoolers’ creative personality. This direct association remained robust after accounting for autonomy, indicating that everyday family practices make an independent contribution to individual differences in creative personality in early childhood.

Our findings extend previous work on family influences on children’s creativity that has mainly focused on family innovation climate and parent–child interaction [20, 45]by highlighting the specific role of structured, predictable, and emotionally supportive family routines. In line with the notion that family routines help maintain a stable family life and harmonious emotional atmosphere [26], the present results suggest that positive routines provide not only structure but also a secure emotional base for exploration and experimentation, rather than merely imposing constraints. In such contexts, preschoolers are more likely to feel safe trying new activities and persisting in the face of minor frustrations, which supports the development of creative personality traits such as curiosity, risk taking, and openness to experience [9, 10, 19].

Importantly, our findings also speak to the long‑standing debate about whether routines and creativity are inherently in tension. Some studies have emphasized that rigid rule enforcement and highly constraining routines may inhibit children’s spontaneous exploration and creative thinking [30, 31, 33]. Our results are more consistent with recent perspectives that distinguish between constraining routines and positive, autonomy‑supportive routines [26, 35, 37]. Within this framework, routines can be understood not as the opposite of creativity, but as a contextual structure that—when organized in a flexible and child‑inclusive manner—supports creative personality development by providing predictable frameworks for behavior without restricting how children think about or approach everyday activities.

From an applied perspective, these findings suggest that parents and caregivers should pay attention not only to whether routines exist, but also to how they are implemented. Clarifying the responsibilities of family members, maintaining predictable daily arrangements, and providing emotionally warm interactions, while at the same time allowing preschoolers appropriate freedom and voice within those routines, appear to be conducive to the development of creative personality. This balance between structure and flexibility may be particularly important in sociocultural contexts that place a strong emphasis on rules and collective harmony [17, 30, 31].

### The mediating role of autonomy in the association between family routines and creative personality

Beyond the direct association, a key contribution of this study is the finding that autonomy partially mediates the link between positive family routines and preschoolers’ creative personality. In our mediation model, positive family routines significantly predicted higher autonomy, which in turn significantly predicted higher creative personality, and the indirect pathway from positive family routines → autonomy → creative personality accounted for a substantial proportion of the total effect. This pattern provides empirical support for our developmental hypothesis that autonomy serves as a psychological bridge connecting family routines and creative personality.

From the perspective of ecological systems theory, Bronfenbrenner [7]emphasized that children’s development is shaped by proximal processes within nested ecological systems, with the family serving as a central microsystem. Our findings are consistent with this view, showing that everyday family routines—an important component of proximal processes—are systematically related to children’s autonomy and, indirectly, to their creative personality. When family routines are predictable and emotionally supportive, they provide repeated opportunities for children to participate in daily decision‑making, to take responsibility for age‑appropriate tasks, and to negotiate roles and expectations with caregivers. These experiences, in turn, foster behavioral, emotional, and cognitive autonomy [27, 37, 38, 46].

Self‑Determination Theory further posits that environmental factors influence development by shaping individuals’ sense of autonomy and self‑determination [41]. The present results extend this theoretical framework to early childhood by showing that autonomy is not only a desirable developmental outcome, but also a key mechanism through which positive family environments are translated into creative personality. In families with positive routines, parents are more likely to provide a structured but autonomy‑supportive context, acknowledging children’s perspectives, offering meaningful choices, and minimizing controlling pressures [26, 41]. Such contexts may enhance children’s intrinsic motivation and willingness to explore, which are central to creative engagement [11, 13, 40].

Our findings also align with research showing that autonomy is a core component of creative personality [39, 40]. Preschoolers with higher autonomy tend to demonstrate stronger initiative, self‑regulation, and persistence [24, 38], and are less dependent on external approval when expressing their ideas [47]. These characteristics are closely related to the “maverick” aspects of creative personality described in previous work [39, 48]. The observed mediating effect suggests that when positive family routines promote the development of autonomy, they simultaneously lay a foundation for the emergence of these creative personality traits.

Taken together, the mediation model verified in this study indicates that positive family routines and children’s autonomy jointly contribute to the formation of creative personality in early childhood. This pattern differs from prior research that has primarily examined direct links between parenting styles or parent–child interaction and creativity [19, 20, 45, 49], by specifying a theoretically grounded mechanism (autonomy) through which everyday routines exert their influence. From an intervention standpoint, if the goal is to enhance preschoolers’ creative personality, simply increasing the number of activities or routines may be insufficient; instead, it is crucial to design routines that explicitly support children’s autonomous participation and decision‑making.

### Theoretical and practical implications

The present findings have several important theoretical and practical implications. Theoretically, they enrich ecological and motivational accounts of creativity development by demonstrating that positive family routines—conceptualized as structured, emotionally warm, and jointly endorsed daily patterns—are linked to preschoolers’ creative personality both directly and indirectly through autonomy. This underscores that creative personality in early childhood is not only an individual disposition [9, 10, 19], but also an outcome of ongoing person–environment transactions within the family microsystem [7, 17, 26].

Moreover, our results contribute to a more nuanced understanding of the role of rules and routines in creativity. Rather than viewing rules as purely inhibitory, the current study suggests that when they are embedded in positive routines, rules can provide a secure framework within which children safely “break” or flexibly reinterpret conventions at the level of ideas and strategies. This insight is particularly relevant in cultural contexts that place strong emphasis on compliance and rule‑following [30, 31, 33, 34], indicating that supporting creativity does not necessarily require abandoning structure, but rather transforming structure into an autonomy‑supportive, dialogical process.

Practically, the findings provide guidance for families, early childhood educators, and policy makers. For parents, establishing predictable, stable, and emotionally supportive routines—such as shared reading times, collaborative play, and family discussions—while inviting children’s input (e.g., choosing games, proposing topics, helping plan small celebrations) may simultaneously strengthen autonomy and creative personality. For kindergartens, family–school cooperation programs could include components that help caregivers reflect on their routines, identify constraining patterns, and redesign them into more autonomy‑supportive practices. At the policy level, early childhood education guidelines might explicitly emphasize the dual importance of structured family life and children’s autonomy for fostering creativity, encouraging community‑based interventions that support parents in building positive family routines.

Finally, considering that personality characteristics formed in the preschool stage can set the tone for later behavioral patterns, physical health, and subjective well‑being [22, 23], promoting creative personality through positive family routines and autonomy has potential benefits that extend far beyond creativity itself. Early interventions that support families in constructing such routines may therefore represent a cost‑effective avenue for enhancing children’s long‑term developmental trajectories.

### Limitations and future research directions

This study still has several limitations that should be acknowledged. First, the cross‑sectional design limits our ability to draw definitive causal inferences about the directionality of effects between positive family routines, autonomy, and creative personality. To address this limitation, future research should adopt longitudinal and multi‑wave designs across different regions and cultural contexts, so as to test developmental trajectories and strengthen the generalizability of the findings.

Second, parents’ evaluations were used to measure preschoolers’ autonomy and creative personality, which may lead to systematic positive bias and shared‑method variance. Although our statistical test suggested that common‑method bias was not severe, future research should incorporate multiple informants (e.g., kindergarten teachers, peers) and multi‑method assessments (e.g., behavioral observations, performance‑based creativity tasks) to obtain more objective and comprehensive indicators [19, 20, 27, 38, 49]. Given that kindergartens are also key settings for preschoolers’ daily life, future studies could integrate classroom observations and teacher ratings to examine how family routines and school routines jointly shape children’s autonomy and creative personality.

Third, although the instruments used in this study showed acceptable reliability and validity, parent‑report scales may not capture the full complexity of young children’s autonomous behavior and everyday creativity. Future research could employ experimental and naturalistic paradigms (e.g., problem‑solving tasks, free‑play settings, or structured creative activities) to assess autonomy‑related behaviors and creative expressions more directly, and to test how children respond to experimentally manipulated routine structures.

Finally, it would be valuable to examine which specific components of positive family routines (e.g., educational, leisure, celebratory, or extended‑family interaction routines) [26, 37] are most strongly linked to creative personality, and whether these associations vary by child characteristics such as temperament or gender [19, 24, 45]. Such work would help translate the present findings into more fine‑grained, evidence‑based recommendations for designing family routines that optimally support the development of creative personality in early childhood.

## Conclusion

This study provides empirical evidence that positive family routines are associated with preschoolers’ creative personality, both directly and indirectly through the mediating role of child autonomy. Grounded in ecological systems theory and self-determination theory, our findings suggest that positive family routines—characterized by predictability, emotional warmth, and child-inclusive participation—foster autonomy, which in turn cultivates creative dispositions such as curiosity, risk-taking, and persistence. The partial mediation highlights autonomy as a key developmental bridge linking everyday family practices to early creative potential.

These findings carry practical implications for parents, educators, and policymakers. Cultivating creativity in early childhood requires transforming family routines into autonomy-supportive practices that balance structure with opportunities for child agency. By specifying how family microsystems contribute to creative personality formation, this study advances developmental understanding and provides a theoretical basis for family-based interventions during the preschool years.

## Supplementary Information

Supplementary Material 1.

Supplementary Material 2.

Supplementary Material 3.

## Data Availability

The datasets generated and analyzed during the current study are not publicly available due to ethical restrictions (participant confidentiality) but may be available from the corresponding author upon reasonable request.

## Abbreviations

CAP: Creativity Assessment Packet
CFA: Confirmatory factor analysis
CFI: Comparative fit index
CI: Confidence interval
FRS: Family Routines Scale
IRB: Institutional Review Board
RMSEA: Root mean square error of approximation
SD: Standard deviation
SDT: Self‑Determination Theory
SE: Standard error
SEM: Structural equation modeling
SRMR: Standardized root mean square residual
TLI: Tucker–Lewis index

## References

[1] Gilhooly K. Creativity: definitions and computability. J Cogn Psychol. 2025;1–11. 10.1080/20445911.2024.2449028.

[2] Runco MA. Updating the Standard Definition of Creativity to Account for the Artificial Creativity of AI. Creativity Res J. 2025;37(1):1–5. 10.1080/10400419.2023.2257977.

[3] Giancola M, Palmiero M, Bocchi A, Piccardi L, Nori R, D’Amico S. Divergent thinking in Italian elementary school children: The key role of probabilistic reasoning style. Cogn Process. 2022;23(4):637–45. 10.1007/s10339-022-01104-2. 35881317 10.1007/s10339-022-01104-2

[4] Giancola M, Palmiero M, Pino MC, Sannino M, D’Amico S. How do children think outside the box? Fluid intelligence and divergent thinking: A moderated mediation study of field dependent-independent cognitive style and gender. Children. 2024;11(1):89. 10.3390/children11010089. 38255402 10.3390/children11010089 PMC10814549

[5] Rhodes M. An analysis of creativity. Phi Delta Kappan. 1961;42(7):305–10.

[6] Sternberg RJ, Lubart TI. An investment theory of creativity and its development. Hum Dev. 1991;34(1):1–31. 10.1159/000277029.

[7] Bronfenbrenner U. The ecology of human development. Cambridge, MA: Harvard University Press; 1979.

[8] Guilford JP. The nature of human intelligence. New York: McGraw-Hill; 1967.

[9] Lin C, D. Creative personality·Creative education·Creative learning. J Chin Soc Educ. 2000;1:5–8. 10.3969/j.issn.1002-4808.2000.01.002.

[10] Wei C, L., Lu R, P. A Review of Creativity and Creative Personality. Res Mod Basic Educ. 2020;39(3):137–44.

[11] Conner T, DeYoung S, C, G., Silvia P, J. Everyday creative activity as a path to flourishing. J Posit Psychol. 2018;13(2):181–9. 10.1080/17439760.2016.1257049.

[12] Karwowski M, Lebuda I, Szumski G, Firkowska-Mankiewicz A. From moment-to-moment to day-to-day: Experience sampling and diary investigations in adults’ everyday creativity. Psychol Aesthet Creativity Arts. 2017;11(3):309–24. 10.1037/aca0000127.

[13] Silvia PJ, Beaty RE, Nusbaum EC, Eddington KM, Levin-Aspenson H, Kwapil TR. Everyday creativity in daily life: An experience-sampling study of little c creativity. Psychol Aesthet Creativity Arts. 2014;8(2):183–8. 10.1037/a0035722.

[14] Khalifaeva O, A. Relationship of Creativity and Cognitive Styles in Early Adulthood. Siberian J Psychol. 2018;69:172–90. 10.17223/17267080/69/10.

[15] Glăveanu VP. Paradigms in the study of creativity: Introducing the perspective of cultural psychology. New Ideas Psychol. 2010;28(1):79–93. 10.1016/j.newideapsych.2009.07.007.

[16] Lubart TI. Creativity across cultures. Sternberg, editor, Handbook of creativity. Cambridge, UK: Cambridge University Press; 1999. pp. 339–50.

[17] Kagitcibasi C. Autonomy and Relatedness in Cultural Context. J Cross-Cult Psychol. 2005;36(4):403–22. 10.1177/0022022105275959.

[18] Li X, Liu Y, X, X., Shen J, L. The Relation between Adolescents’ Creative Personality and Creativity: Evidence from Comparisons of Adolescents in American and China. Psychol Explor. 2014;34(2):186–92. 10.3969/j.issn.1003-5184.2014.02.015.

[19] Lee KH. The relationship between creative thinking ability and creative personality of preschoolers. Int Educ J. 2005;6(2):194–9.

[20] Shu Z, He Q, Li X, Zhang M, Zhang J, Y, H., Fang X, Y. Effect of Maternal Stress on Preschoolers’ Creative Personality: The Mediating Role of Mothers’ Parenting Styles. Psychol Dev Educ. 2016;32(3):276–84. / j. cnki. issn1001-4918. 2016.03.03.

[21] Roberts BW, DelVecchio WF. The rank-order consistency of personality traits from childhood to old age: A quantitative review of longitudinal studies. Psychol Bull. 2000;126(1):3–25. 10.1037/0033-2909.126.1.3. 10668348 10.1037/0033-2909.126.1.3

[22] Chen H, M., Mo L. The Structure and Cultivation of Preschool Children’s Scientific and Innovative Personality. J Mod Educ. 2005;01:49–52. doi: CNKI: SUN: XDJY.0.2005-01-012.

[23] Pekhota G. Psychological and pedagogical conditions for the formation of an intellectual and creative personality. Fundamental Appl Researches Pract Lead Sci Schools. 2018;27(3):165–9. 10.33531/farplss.2018.3.18.

[24] Zou X, Y. On the Characteristics of Autonomy Development in Children of 3 ~ 5 Years. Early Child Educ (Educational Sciences). 2006;12:38–42. 10.3969/j.issn.1004-4604-B.2006.12.011.

[25] Hennessey BA, Amabile TM. Creativity. Ann Rev Psychol. 2010;61:569–98. 10.1146/annurev.psych.093008.100416. 19575609 10.1146/annurev.psych.093008.100416

[26] Spagnola M, Fiese BH. Family routines and rituals: A context for development in the lives of young children. Infants Young Child. 2007;20(4):284–99. 10.1097/01.IYC.0000290352.32170.5a.

[27] Forman DR. Autonomy, compliance, and internalization. In: Brownell CA, Kopp CB, editors. Socioemotional development in the toddler years: Transitions and transformations. New York, NY: Guilford Press; 2007. pp. 285–319.

[28] Bandura A. Social foundations of thought and action: A social cognitive theory. Englewood Cliffs, NJ: Prentice-Hall; 1986.

[29] Jensen EW, James SA, Boyce WT, Hartnett SA. The family routines inventory: Development and validation. Social Sci Med. 1983;17:201–11. 10.1016/0277-9536(83)90117-X. 10.1016/0277-9536(83)90117-x 6844952

[30] Bayanova L, F., Mustafin T, R. Factors of compliance of a child with rules in a Russian cultural context. Eur Early Child Educ Res J. 2016;24(3):357–64. 10.1080/1350293X.2016.1164394.

[31] Bayanova L, F., Chulyukin K, S. The impact of cultural congruence on the creative thinking of primary school children. Psychol Russia: State Art. 2018;11(1):61–70. 10.11621/pir.2018.0105.

[32] Cohendet P, Llerena P, Simon L. The Routinization of Creativity: Lessons From the Case of a Video-game Creative Powerhouse. Jahrbucher Fur Nationalokonomie Und Statistik. 2014;234(2–3):120–41. 10.1515/9783110509205-002.

[33] Gong Y, Liu J. On Training Children’s Consciousness and Behaviors of Rules. Stud Preschool Educ. 2009;1:69–71. doi: CNKI: SUN: XQJY.0.2009-01-019.

[34] Guo Y, L., Tang B, M. Family Education on the Cultivation of Primary and Middle School Students’ Consciousness and Behavior of Rules. Moral Educ China. 2015;7:29–32. doi: CNKI: SUN: DEYU.0.2015-07-016.

[35] Koome F, Hocking C, Daniel S. Why Routines Matter: The Nature and Meaning of Family Routines in the Context of Adolescent Mental Illness. J Occup Sci. 2012;19(4):312–25. 10.1080/14427591.2012.718245.

[36] Lebuda I, Jankowska D, M., Karwowski M. Parents’ Creative Self-Concept and Creative Activity as Predictors of Family Lifestyle. Int J Environ Res Public Health. 2020;17:9558–75. 10.3390/ijerph17249558. 33371220 10.3390/ijerph17249558 PMC7766455

[37] Zhang F. The correlative study between family routines and 4–year–old children’s autonomy (Master’s thesis). Liaoning Normal University, Dalian, China. 2010.

[38] Hei LJ. A study on structure and development characteristic of autonomy of 3 to 5-year-old children (Dissertation). Liaoning Normal University, Dalian, China. 2008.

[39] Kirsch C, Lubart T, Houssemand C. Creative personality profile in social science: The leading role of autonomy. Creativity Theory–Research–Applications. 2015;2(2):180–211. 10.1515/ctra-2015-0020.

[40] Wang ZY, Dong SY. Autonomy as core of creativity and compliance: Moderated moderation model of maternal parenting behaviors. Creativity Res J. 2019;31(1):74–82. 10.1080/10400419.2019.1577674.

[41] Deci EL, Ryan RM. The support of autonomy and the control of behavior. J Personal Soc Psychol. 1987;53(6):1024–37. 10.1037/0022-3514.53.6.1024. 10.1037//0022-3514.53.6.1024 3320334

[42] Bellieni C. Creativity in Children Growing Up in Dysfunctional Families. Psychol Stud. 2025;1–7. 10.1007/s12646-024-00816-z.

[43] Yan Y, Zhang Z, J, Y., Dong S, H. Influence of Childhood Family Routines on Adult Depression: A Cross Sectional Study. Front Psychol. 2021;12:654433. 10.3389/fpsyg.2021.654433y. 34290646 10.3389/fpsyg.2021.654433 PMC8288247

[44] Lin X, T., Wang M, R. Williams Handbook on Creative Thinking Activities. Taipei: Psychology; 1997.

[45] Kim JH, Lee KN. The Modulating Effects of Mother-Child Interaction on the Relation between Preschooler’s Temperament and Creative Personality. J Learner-Centered Curriculum Instruction. 2020;20(3):841–66. 10.22251/jlcci.2020.20.3.841.

[46] Sotés-Elizalde MÁ, Urpi C. Family Involvement, Autonomy, and Social Competency in Homeschooling. Educ United States China. 2015;5(11):714–23. 10.17265/2161-6248/2015.11.003.

[47] Wink P. Self- and object-directness in adult women. J Pers. 1991;59:769–91. 10.1111/1467-6494.ep9202104579. 1774619 10.1111/j.1467-6494.1991.tb00931.x

[48] Giovacchini PL. The creative person as maverick. New York: Guilford Press; 1991. 10.1521/jaap.1.1991.19.2.174 1938581

[49] Deng X. P. Relationship between teachers’ behavior, parent-child interaction and children’s creativity in preschool (Master’s thesis). Northeast Normal University, Changchun, China. 2013

