# Supplementary material for: The formation of preschooler’s creative personality: the promotion mechanism of positive family routines
Source: BMC Psychol. 2026 Apr 9;14:717. doi: 10.1186/s40359-026-04506-5 (PMC13173852; doi:10.1186/s40359-026-04506-5)
Supplement: Supplementary file 2 — Supplementary Material 2. [file 40359_2026_4506_MOESM2_ESM.docx]

**附录—研究中使用的问卷（中文原版）**

## 家庭规则量表

尊敬的家长：

您好！本调查旨在了解幼儿家庭日常生活中的一些基本情况，为家庭教育研究提供科学依据。请您根据家庭的真实情况，逐题阅读并在最符合您家庭实际情形的选项上打“√”。所有回答无对错之分，结果仅用于学术研究，我们将严格保密。感谢您的支持与合作！

作答说明：每题均有五个选项，“1”代表“完全不符合”，“2”代表“基本不符合”，“3”代表“一般”，“4”代表“基本符合”，“5”代表“完全符合”。请根据您家庭的实际情况，选择最接近的描述。

| **序号** | **题 目** | **完全**  **不符** | **基本**  **不符** | **一般** | **基本**  **符合** | **完全**  **符合** |
| --- | --- | --- | --- | --- | --- | --- |
| 1 | 每当家里有人过生日时，都会互相提醒 | 1 | 2 | 3 | 4 | 5 |
| 2 | 家里大多数事情都会全家一起讨论 | 1 | 2 | 3 | 4 | 5 |
| 3 | 家人经常和亲戚来往 | 1 | 2 | 3 | 4 | 5 |
| 4 | 每周末全家都会一起去一些地方 | 1 | 2 | 3 | 4 | 5 |
| 5 | 父母每天都会和孩子做一些户外活动，如购物、散步、玩耍等 | 1 | 2 | 3 | 4 | 5 |
| 6 | 我家和亲戚家常常彼此相互关照 | 1 | 2 | 3 | 4 | 5 |
| 7 | 每周都有一个特定的“家庭时间”，全家人一起度过 | 1 | 2 | 3 | 4 | 5 |
| 8 | 孩子向父母询问问题时，父母会尽量解答 | 1 | 2 | 3 | 4 | 5 |
| 9 | 每个周末都会有一些计划和讨论，如下周的安排、本周的总结等 | 1 | 2 | 3 | 4 | 5 |
| 10 | 每周家里人都会安排好一起去一些地方 | 1 | 2 | 3 | 4 | 5 |
| 11 | 每周末家人都会一起去购物、或去其它地方 | 1 | 2 | 3 | 4 | 5 |
| 12 | 亲戚朋友来我家时，我总让我的孩子向他们问好 | 1 | 2 | 3 | 4 | 5 |
| 13 | 父/母或父母双方下班回家后都要抽时间和孩子玩 | 1 | 2 | 3 | 4 | 5 |
| 14 | 每年每个人的生日都会庆祝 | 1 | 2 | 3 | 4 | 5 |
| 15 | 父母经常和孩子做一些游戏 | 1 | 2 | 3 | 4 | 5 |
| 16 | 家里有人出门或回家时，家人会彼此留意并关注 | 1 | 2 | 3 | 4 | 5 |
| 17 | 父/母或父母双方每天都有一些时间照顾孩子 | 1 | 2 | 3 | 4 | 5 |
| 18 | 每天父/母或父母双方都会给孩子读故事或讲故事 | 1 | 2 | 3 | 4 | 5 |
| 19 | 家里有重要的事情都会和亲戚商量 | 1 | 2 | 3 | 4 | 5 |
| 20 | 父母每天都有一些时间专门和孩子说话 | 1 | 2 | 3 | 4 | 5 |
| 21 | 父母的结婚纪念周年每年都会全家人一起庆祝 | 1 | 2 | 3 | 4 | 5 |

## 自主性发展量表

以下调查旨在了解幼儿在日常生活中表现出的自主性行为。请您根据孩子的真实情况，逐题阅读并在最符合的选项上打“√”。所有信息仅用于学术研究，我们将严格保密。感谢您的配合！

作答说明：每题均有五个选项，“1”代表“从来不”，“2”代表“几乎不”，“3”代表“偶尔”，“4”代表“有时”，“5”代表“经常”。请根据您孩子的实际表现，选择最合适的选项。

| **序号** | **题项** | **从来不** | **几乎不** | **偶尔** | **有时** | **经常** |
| --- | --- | --- | --- | --- | --- | --- |
| 1 | 能自己收拾书包 | 1 | 2 | 3 | 4 | 5 |
| 2 | 自己能穿上长裤 | 1 | 2 | 3 | 4 | 5 |
| 3 | 能清楚地向父母说出自己的请求 | 1 | 2 | 3 | 4 | 5 |
| 4 | 心情不好时缠着大人 | 1 | 2 | 3 | 4 | 5 |
| 5 | 想做的事不被允许时，会哭闹 | 1 | 2 | 3 | 4 | 5 |
| 6 | 说不清楚自己的想法 | 1 | 2 | 3 | 4 | 5 |
| 7 | 受到家长责备时会哭闹 | 1 | 2 | 3 | 4 | 5 |
| 8 | 能自己照顾小动物或植物 | 1 | 2 | 3 | 4 | 5 |
| 9 | 被小朋友抢玩具后会哭 | 1 | 2 | 3 | 4 | 5 |
| 10 | 要看电视而家长不让他/她看时，会哭闹 | 1 | 2 | 3 | 4 | 5 |
| 11 | 希望自己决定上哪个特长班 | 1 | 2 | 3 | 4 | 5 |
| 12 | 老师布置的事能较完整地告诉父母 | 1 | 2 | 3 | 4 | 5 |
| 13 | 能自己叠被子 | 1 | 2 | 3 | 4 | 5 |
| 14 | 能使用筷子吃饭 | 1 | 2 | 3 | 4 | 5 |
| 15 | 画画时，总要成人在旁边陪着 | 1 | 2 | 3 | 4 | 5 |
| 16 | 家长说错话时能给家长指出来 | 1 | 2 | 3 | 4 | 5 |
| 17 | 和父母交谈时，能发表自己的意见 | 1 | 2 | 3 | 4 | 5 |
| 18 | 想吃零食而被大人制止时，会生气 | 1 | 2 | 3 | 4 | 5 |
| 19 | 能独立穿上外套，拉好拉链或扣好扣子 | 1 | 2 | 3 | 4 | 5 |
| 20 | 做手工时，总是让成人在一旁帮助 | 1 | 2 | 3 | 4 | 5 |
| 21 | 能清楚地向小伙伴表达自己的想法 | 1 | 2 | 3 | 4 | 5 |
| 22 | 能自己挤牙膏刷牙 | 1 | 2 | 3 | 4 | 5 |

## 创造性人格量表

以下调查旨在了解幼儿在日常生活中的一些行为倾向和兴趣表现。请您根据孩子的真实情况，逐题阅读并在最符合的选项上打“√”。所有回答无对错之分，结果仅用于学术研究，我们将严格保密。感谢您的支持！

作答说明：每题均有三个选项，“1”代表“不像”，“2”代表“一般”，“3”代表“很像”。请根据您孩子的实际表现，选择最接近的描述。

| **序号** | **题项** | **不像** | **一般** | **很像** |
| --- | --- | --- | --- | --- |
| 1 | 在学校里，喜欢试着对事情或问题作猜测，即使不一定都猜对也无所谓。 | 1 | 2 | 3 |
| 2 | 喜欢仔细观察没有看过的东西，以了解详细的情形。 | 1 | 2 | 3 |
| 3 | 喜欢听变化多端和富有想象力的故事。 | 1 | 2 | 3 |
| 4 | 画图时喜欢临摹别人的作品。 | 1 | 2 | 3 |
| 5 | 喜欢利用旧报纸、旧日历及旧罐头等废物做成各种好玩的东西。 | 1 | 2 | 3 |
| 6 | 喜欢幻想一些想知道或想做的事。 | 1 | 2 | 3 |
| 7 | 如果事情不能一次完成，会继续尝试，直到成功为止。 | 1 | 2 | 3 |
| 8 | 做功课时喜欢参考各种不同的资料，以便得到多方面的了解。 | 1 | 2 | 3 |
| 9 | 喜欢用相同的方法做事情，不愿去找其他新的方法。 | 1 | 2 | 3 |
| 10 | 喜欢探究事情的真假。 | 1 | 2 | 3 |
| 11 | 喜欢做许多新鲜的事。 | 1 | 2 | 3 |
| 12 | 不喜欢交新朋友。 | 1 | 2 | 3 |
| 13 | 喜欢想一些不会在他/她身上发生过的事情。 | 1 | 2 | 3 |
| 14 | 喜欢想象有一天能成为艺术家、音乐家或诗人。 | 1 | 2 | 3 |
| 15 | 会因为一些令人兴奋的念头而忘记了其他的事。 | 1 | 2 | 3 |
| 16 | 宁愿生活在太空站，也不喜欢住在地球上。 | 1 | 2 | 3 |
| 17 | 认为所有的问题都有固定的答案。 | 1 | 2 | 3 |
| 18 | 喜欢与众不同的事情。 | 1 | 2 | 3 |
| 19 | 常想要知道别人正在想什么。 | 1 | 2 | 3 |
| 20 | 喜欢故事或电视节目所描写的事。 | 1 | 2 | 3 |
| 21 | 喜欢和朋友一起和他们分享他/她的想法。 | 1 | 2 | 3 |
| 22 | 如果一本故事书的最后一页被撕掉了，他/她会自己编造一个故事，把结局补上去。 | 1 | 2 | 3 |
| 23 | 长大后想做一些别人从没想过的事情。 | 1 | 2 | 3 |
| 24 | 尝试新的游戏和活动，是一件有趣的事。 | 1 | 2 | 3 |
| 25 | 不喜欢太多的规则限制。 | 1 | 2 | 3 |
| 26 | 喜欢解决问题，即使没有正确的答案也没关系。 | 1 | 2 | 3 |
| 27 | 有许多事情都很想亲自去尝试。 | 1 | 2 | 3 |
| 28 | 喜欢唱没有人知道的新歌。 | 1 | 2 | 3 |
| 29 | 不喜欢在班上同学面前发表意见。 | 1 | 2 | 3 |
| 30 | 当读小说或看电视时，喜欢把自己想成故事中的人物。 | 1 | 2 | 3 |
| 31 | 喜欢幻想200年前人类生活的情形。 | 1 | 2 | 3 |
| 32 | 常想自己编一首新歌。 | 1 | 2 | 3 |
| 33 | 喜欢翻箱倒柜，看看有些什么东西在里面。 | 1 | 2 | 3 |
| 34 | 画图时，很喜欢改变各种东西的颜色和形状。 | 1 | 2 | 3 |
| 35 | 不敢确定对事情的看法都是对的。 | 1 | 2 | 3 |
| 36 | 对于一件事情先猜猜看，然后再看是不是猜对了，认为这种方法很有趣。 | 1 | 2 | 3 |
| 37 | 认为玩猜谜之类的游戏很有趣。 | 1 | 2 | 3 |
| 38 | 对机器有兴趣，也很想知道它里面是什么样子，以及它是怎样转动的。 | 1 | 2 | 3 |
| 39 | 喜欢可以拆开来的玩具。 | 1 | 2 | 3 |
| 40 | 喜欢想一些新点子，即使用不着也无所谓。 | 1 | 2 | 3 |
| 41 | 认为一篇好的文章应该包含许多不同的意见或观点。 | 1 | 2 | 3 |
| 42 | 为将来可能发生的问题找答案，是一件令人兴奋的事。 | 1 | 2 | 3 |
| 43 | 喜欢尝试新的事物，目的只是为了想知道会有什么结果。 | 1 | 2 | 3 |
| 44 | 玩游戏时，通常是有兴趣参加，而不在乎输赢。 | 1 | 2 | 3 |
| 45 | 喜欢想一些别人常常谈过的事情。 | 1 | 2 | 3 |
| 46 | 当看到一张陌生人的照片时，喜欢去猜测他是怎么样一个人。 | 1 | 2 | 3 |
| 47 | 喜欢翻阅书籍及杂志，但只想知道它的内容是什么。 | 1 | 2 | 3 |
| 48 | 不喜欢探寻事情发生的各种原因。 | 1 | 2 | 3 |
| 49 | 喜欢问一些别人没有想到的问题。 | 1 | 2 | 3 |
| 50 | 无论在家里或在学校，总是喜欢做有趣的事。 | 1 | 2 | 3 |

请您核对以上所有题目是否均已作答，感谢您的支持与配合，祝您生活愉快！
