# Supplementary material for: The formation of preschooler’s creative personality: the promotion mechanism of positive family routines
Source: BMC Psychol. 2026 Apr 9;14:717. doi: 10.1186/s40359-026-04506-5 (PMC13173852; doi:10.1186/s40359-026-04506-5)
Supplement: Supplementary file 3 — Supplementary Material 3. [file 40359_2026_4506_MOESM3_ESM.docx]

## **Appendix: Questionnaires Used in the Study (English Translation)**

**Family Routines Scale (FRS)**

Dear Parent,

This survey aims to understand certain aspects of daily family life to provide a scientific basis for research on family education. Please read each statement carefully and mark the response that best reflects your family’s actual situation. There are no right or wrong answers, and all responses will be kept confidential and used solely for academic research purposes. Thank you for your support and cooperation!

**Response Format:**

Each item is rated on a 5-point scale:

1 = “Completely inconsistent”

2 = “Basically inconsistent”

3 = “Neutral”

4 = “Basically consistent”

5 = “Completely consistent”

Please choose the option that most accurately describes your family’s situation.

| **No.** | **Item** | **Completely Inconsistent** | **Basically Inconsistent** | **Neutral** | **Basically Consistent** | **Completely Consistent** |
| --- | --- | --- | --- | --- | --- | --- |
| 1 | Family members remind each other when someone’s birthday is approaching. | 1 | 2 | 3 | 4 | 5 |
| 2 | Most family matters are discussed together by the whole family. | 1 | 2 | 3 | 4 | 5 |
| 3 | Family members regularly keep in touch with relatives. | 1 | 2 | 3 | 4 | 5 |
| 4 | The whole family goes out together to some places every weekend. | 1 | 2 | 3 | 4 | 5 |
| 5 | Parents engage in outdoor activities with their child every day, such as shopping, walking, or playing. | 1 | 2 | 3 | 4 | 5 |
| 6 | Our family and our relatives often help each other out. | 1 | 2 | 3 | 4 | 5 |
| 7 | There is a specific “family time” each week that the whole family spends together. | 1 | 2 | 3 | 4 | 5 |
| 8 | When the child asks questions, parents try their best to answer them. | 1 | 2 | 3 | 4 | 5 |
| 9 | Every weekend, there is some planning and discussion, such as the next week’s schedule or a review of the current week. | 1 | 2 | 3 | 4 | 5 |
| 10 | Each week, family members arrange to go to certain places together. | 1 | 2 | 3 | 4 | 5 |
| 11 | Every weekend, family members go shopping or go out together. | 1 | 2 | 3 | 4 | 5 |
| 12 | When relatives or friends visit our home, I always have my child greet them. | 1 | 2 | 3 | 4 | 5 |
| 13 | After returning home from work, the father, mother, or both parents spend time playing with the child. | 1 | 2 | 3 | 4 | 5 |
| 14 | Every family member’s birthday is celebrated each year. | 1 | 2 | 3 | 4 | 5 |
| 15 | Parents often play games with their child. | 1 | 2 | 3 | 4 | 5 |
| 16 | When someone leaves or returns home, family members notice and pay attention. | 1 | 2 | 3 | 4 | 5 |
| 17 | The father, mother, or both parents spend time each day caring for the child. | 1 | 2 | 3 | 4 | 5 |
| 18 | The father, mother, or both parents read stories or tell stories to the child every day. | 1 | 2 | 3 | 4 | 5 |
| 19 | Important family matters are discussed with relatives. | 1 | 2 | 3 | 4 | 5 |
| 20 | Parents spend time each day specifically talking with their child. | 1 | 2 | 3 | 4 | 5 |
| 21 | The parents’ wedding anniversary is celebrated by the whole family each year. | 1 | 2 | 3 | 4 | 5 |

## **Autonomy of Children Scale**

This survey aims to understand children’s autonomous behaviors in daily life. Please read each statement carefully and mark the response that best reflects your child’s actual behavior. All information will be kept confidential and used solely for academic research purposes. Thank you for your participation!

****Response Format:****
Each item is rated on a 5-point scale:
1 = “Never”
2 = “Rarely”
3 = “Occasionally”
4 = “Sometimes”
5 = “Always”
Please choose the option that most accurately describes your child’s typical behavior.

| **No.** | **Item** | **Never** | **Rarely** | **Occasionally** | **Sometimes** | **Always** |
| --- | --- | --- | --- | --- | --- | --- |
| 1 | Can pack their own school bag. | 1 | 2 | 3 | 4 | 5 |
| 2 | Can put on pants by themselves. | 1 | 2 | 3 | 4 | 5 |
| 3 | Can clearly express their requests to parents. | 1 | 2 | 3 | 4 | 5 |
| 4 | Clings to adults when in a bad mood. | 1 | 2 | 3 | 4 | 5 |
| 5 | Cries or fusses when not allowed to do something they want. | 1 | 2 | 3 | 4 | 5 |
| 6 | Has difficulty expressing their thoughts clearly. | 1 | 2 | 3 | 4 | 5 |
| 7 | Cries or fusses when scolded by parents. | 1 | 2 | 3 | 4 | 5 |
| 8 | Can take care of small animals or plants by themselves. | 1 | 2 | 3 | 4 | 5 |
| 9 | Cries after another child takes away their toy. | 1 | 2 | 3 | 4 | 5 |
| 10 | Cries or fusses when parents do not allow them to watch TV. | 1 | 2 | 3 | 4 | 5 |
| 11 | Prefers to decide for themselves which extracurricular class to take. | 1 | 2 | 3 | 4 | 5 |
| 12 | Can relatively completely inform parents about tasks assigned by the teacher. | 1 | 2 | 3 | 4 | 5 |
| 13 | Can fold their own blanket. | 1 | 2 | 3 | 4 | 5 |
| 14 | Can use chopsticks to eat. | 1 | 2 | 3 | 4 | 5 |
| 15 | Needs an adult to stay with them when drawing. | 1 | 2 | 3 | 4 | 5 |
| 16 | Can point out mistakes when parents say something wrong. | 1 | 2 | 3 | 4 | 5 |
| 17 | Can express their own opinions when talking with parents. | 1 | 2 | 3 | 4 | 5 |
| 18 | Gets angry when an adult stops them from eating snacks. | 1 | 2 | 3 | 4 | 5 |
| 19 | Can independently put on a coat and zip it up or button it. | 1 | 2 | 3 | 4 | 5 |
| 20 | Always needs an adult to assist them when doing crafts. | 1 | 2 | 3 | 4 | 5 |
| 21 | Can clearly express their thoughts to peers. | 1 | 2 | 3 | 4 | 5 |
| 22 | Can put toothpaste on their toothbrush and brush their teeth by themselves. | 1 | 2 | 3 | 4 | 5 |

## **Creative Personality Scale (subscale of the Creativity Assessment Packet, CAP)**

This survey aims to understand children’s behavioral tendencies and interests in everyday situations. Please read each statement carefully and mark the response that best reflects your child’s actual behavior. There are no right or wrong answers, and all responses will be kept confidential and used solely for academic research purposes. Thank you for your support!

**Response Format:**

Each item is rated on a 3-point scale:

1 = “Unlike my child”

2 = “Neutral”

3 = “Like my child”

Please choose the option that most accurately describes your child’s behavior.

| **No.** | **Item** | **Unlike My Child** | **Neutral** | **Like My Child** |
| --- | --- | --- | --- | --- |
| 1 | At school, likes to make guesses about things or problems, even if not always correct. | 1 | 2 | 3 |
| 2 | Likes to carefully observe things they haven’t seen before to understand details. | 1 | 2 | 3 |
| 3 | Likes stories that are varied, imaginative, and full of changes. | 1 | 2 | 3 |
| 4 | Prefers to copy others’ work when drawing. | 1 | 2 | 3 |
| 5 | Likes to make interesting things from old newspapers, calendars, cans, and other recyclable items. | 1 | 2 | 3 |
| 6 | Likes to imagine things they want to know or do. | 1 | 2 | 3 |
| 7 | If a task is not completed at once, will keep trying until successful. | 1 | 2 | 3 |
| 8 | Likes to consult different materials when doing schoolwork to gain a broader understanding. | 1 | 2 | 3 |
| 9 | Prefers to do things the same way and is reluctant to try new methods. | 1 | 2 | 3 |
| 10 | Likes to explore whether things are true or not. | 1 | 2 | 3 |
| 11 | Likes to do many new things. | 1 | 2 | 3 |
| 12 | Does not like making new friends. | 1 | 2 | 3 |
| 13 | Likes to think about things that have never happened to them. | 1 | 2 | 3 |
| 14 | Likes to imagine becoming an artist, musician, or poet someday. | 1 | 2 | 3 |
| 15 | Gets so caught up in exciting ideas that they forget about other things. | 1 | 2 | 3 |
| 16 | Would rather live on a space station than on Earth. | 1 | 2 | 3 |
| 17 | Believes that all problems have fixed answers. | 1 | 2 | 3 |
| 18 | Likes things that are different or unusual. | 1 | 2 | 3 |
| 19 | Often wonders what others are thinking. | 1 | 2 | 3 |
| 20 | Likes things depicted in stories or TV programs. | 1 | 2 | 3 |
| 21 | Likes to share their thoughts with friends. | 1 | 2 | 3 |
| 22 | If the last page of a storybook is torn, will make up their own ending. | 1 | 2 | 3 |
| 23 | Wants to do things that no one has ever thought of when they grow up. | 1 | 2 | 3 |
| 24 | Finds trying new games and activities interesting. | 1 | 2 | 3 |
| 25 | Dislikes having too many rules. | 1 | 2 | 3 |
| 26 | Enjoys solving problems, even if there is no correct answer. | 1 | 2 | 3 |
| 27 | Wants to try many things themselves. | 1 | 2 | 3 |
| 28 | Likes to sing new songs that no one has heard before. | 1 | 2 | 3 |
| 29 | Dislikes expressing opinions in front of classmates. | 1 | 2 | 3 |
| 30 | When reading novels or watching TV, likes to imagine themselves as a character. | 1 | 2 | 3 |
| 31 | Likes to imagine what life was like 200 years ago. | 1 | 2 | 3 |
| 32 | Often thinks about making up a new song. | 1 | 2 | 3 |
| 33 | Likes to rummage through boxes and drawers to see what’s inside. | 1 | 2 | 3 |
| 34 | When drawing, likes to change the colors and shapes of things. | 1 | 2 | 3 |
| 35 | Is not sure if their views on things are always correct. | 1 | 2 | 3 |
| 36 | Finds it interesting to guess about something first and then see if they were right. | 1 | 2 | 3 |
| 37 | Enjoys guessing games and similar activities. | 1 | 2 | 3 |
| 38 | Is interested in machines and wants to know what’s inside and how they work. | 1 | 2 | 3 |
| 39 | Likes toys that can be taken apart. | 1 | 2 | 3 |
| 40 | Likes to come up with new ideas, even if they are not useful. | 1 | 2 | 3 |
| 41 | Believes a good article should include different opinions or viewpoints. | 1 | 2 | 3 |
| 42 | Finds it exciting to find answers to possible future problems. | 1 | 2 | 3 |
| 43 | Likes to try new things just to see what will happen. | 1 | 2 | 3 |
| 44 | When playing games, is usually interested in participating, not just winning or losing. | 1 | 2 | 3 |
| 45 | Likes to think about things that others often talk about. | 1 | 2 | 3 |
| 46 | When seeing a photo of a stranger, likes to guess what kind of person they are. | 1 | 2 | 3 |
| 47 | Likes to browse books and magazines just to know their content. | 1 | 2 | 3 |
| 48 | Dislikes exploring the reasons why things happen. | 1 | 2 | 3 |
| 49 | Likes to ask questions that others haven’t thought of. | 1 | 2 | 3 |
| 50 | Whether at home or at school, always likes to do interesting things. | 1 | 2 | 3 |

**Please verify that you have answered all items before submitting. Thank you for your support and cooperation, and we wish you well.**
